# Difficulties on the access to innovative targeted therapies for lung cancer in Spain

**DOI:** 10.1007/s12094-023-03303-5

**Published:** 2023-08-31

**Authors:** Virginia Calvo, Carlos Camps, Enric Carcereny, Manuel Cobo, Manuel Dómine, María Rosario García Campelo, José Luis González Larriba, María Guirado, Florentino Hernando-Trancho, Bartomeu Massutí, Ernest Nadal, Delvys Rodríguez-Abreu, Alfredo Sánchez, Ivana Gabriela Sullivan, Mariano Provencio, the Spanish Lung Cancer Group (SLCG/GECP)

**Affiliations:** 1 Oncology Department, Hospital Universitario Puerta de Hierro Majadahonda, Manuel de Falla 1, Majadahonda, 28222 Madrid, Spain; 2 Hospital General Universitario, Valencia, Spain; 3 Institut Català d’Oncologia, Badalona, Spain; 4 Hospital Regional Universitario, Málaga, Spain; 5 Hospital Universitario Fundación Jiménez Díaz, IIS-FJD, Madrid, Spain; 6 Complexo Hospitalario Universitario A Coruña, A Coruña, Spain; 7 Hospital Clínico San Carlos, Madrid, Spain; 8 Hospital General de Elche, Alicante, Spain; 9 Hospital Clínico San Carlos, Madrid, Spain; 10 Hospital General Universitario Dr. Balmis, Alicante, Spain; 11 Institut Català d’Oncologia, l’Hospitalet de Llobregat, Barcelona, Spain; 12 Hospital Universitario Insular de Gran Canaria, Las Palmas, Spain; 13 Consorcio Hospital Provincial, Castellón, Spain; 14 Hospital Santa Creu i Sant Pau, Barcelona, Spain

**Keywords:** Medicine access, Barriers, Inequity, Innovative treatments, NSCLC, Spain

## Abstract

**Purpose:**

Spanish Lung Cancer Group (SLCG) conducted a review to analyze the barriers to access to innovative targeted therapies for non-small cell lung cancer (NSCLC) in clinical practice in Spain.

**Methods:**

Review all relevant content published on websites of European Commission, European Medicines Agency, and Spanish Agency of Medicines and Medical Products regarding the authorization and access to oncology treatments.

**Results:**

More than 20 targeted therapies are available to treat different molecular alterations in patients with NSCLC. European Commission has approved treatments for genomic alterations involving the following genes: *ALK, RET, ROS1, EGFR, BRAF, NTRK, KRAS, MET*. However, the availability of these therapies in Spain is not complete, as innovative treatments are not reimbursed or funded late, with only five of these alterations currently covered by National Health System.

**Conclusion:**

SLCG considers imperative to improve the access in Spain to innovative treatments for NSCLC to reduce inequity across European countries.

## Introduction

Lung cancer is the second leading cancer diagnosis in the United States for men (12%) or women (13%). It is the most common cause of death, accounting for 21% of estimated deaths in both sexes [1]. According to the Spanish Network of Cancer Registries (REDECAN), it is estimated that more than 30,000 new cases of lung cancer will be detected in Spain in 2023, 22,266 in men and 9016 in women, making it the third most common cancer in Spain [2].

In general, there has been a marked decrease in cancer mortality in Spain in recent decades. In the case of lung cancer, it remains the main cause of cancer death. In 2021, more than 22,000 people died of lung cancer. Mortality will increase in women, mainly due to the later incorporation into smoking [2].

There are two types of lung cancer: small cell lung cancer (SCLC) and non-small cell lung cancer (NSCLC), 80–85% [3].

Historically, NSCLC patients were treated with cytotoxic therapies. With the introduction of targeted and immune therapies in the last decade, there has been a dramatic improvement in survival outcomes in advanced NSCLC. Numerous oncogenic alterations have been identified in NSCLC which are considered actionable [4]. Currently, more than 20 targeted therapies are available to treat different molecular alterations across 8 genes in patients with NSCLC. The European Medical Agency (EMA) approved treatments for these molecular alterations: anaplastic lymphoma kinase (*ALK*) gene arrangements*,* rearranged during transfection (*RET*)*,* ROS proto-oncogene 1 receptor tyrosine kinase (*ROS1*) rearrangements*,* epidermal growth factor receptor (*EGFR*) mutations*, EGFR* exon 20 insertions, B-RAF proto-oncogene serine/threonine kinase (*BRAF*) V600E, neurotrophic tyrosine receptor kinase rearrangements (*NTRK*)*,* Kirsten rat sarcoma viral oncogene (*KRAS*)*,* mesenchymal–epithelial transition (*MET*) [4, 5].

**Fig. 1:**
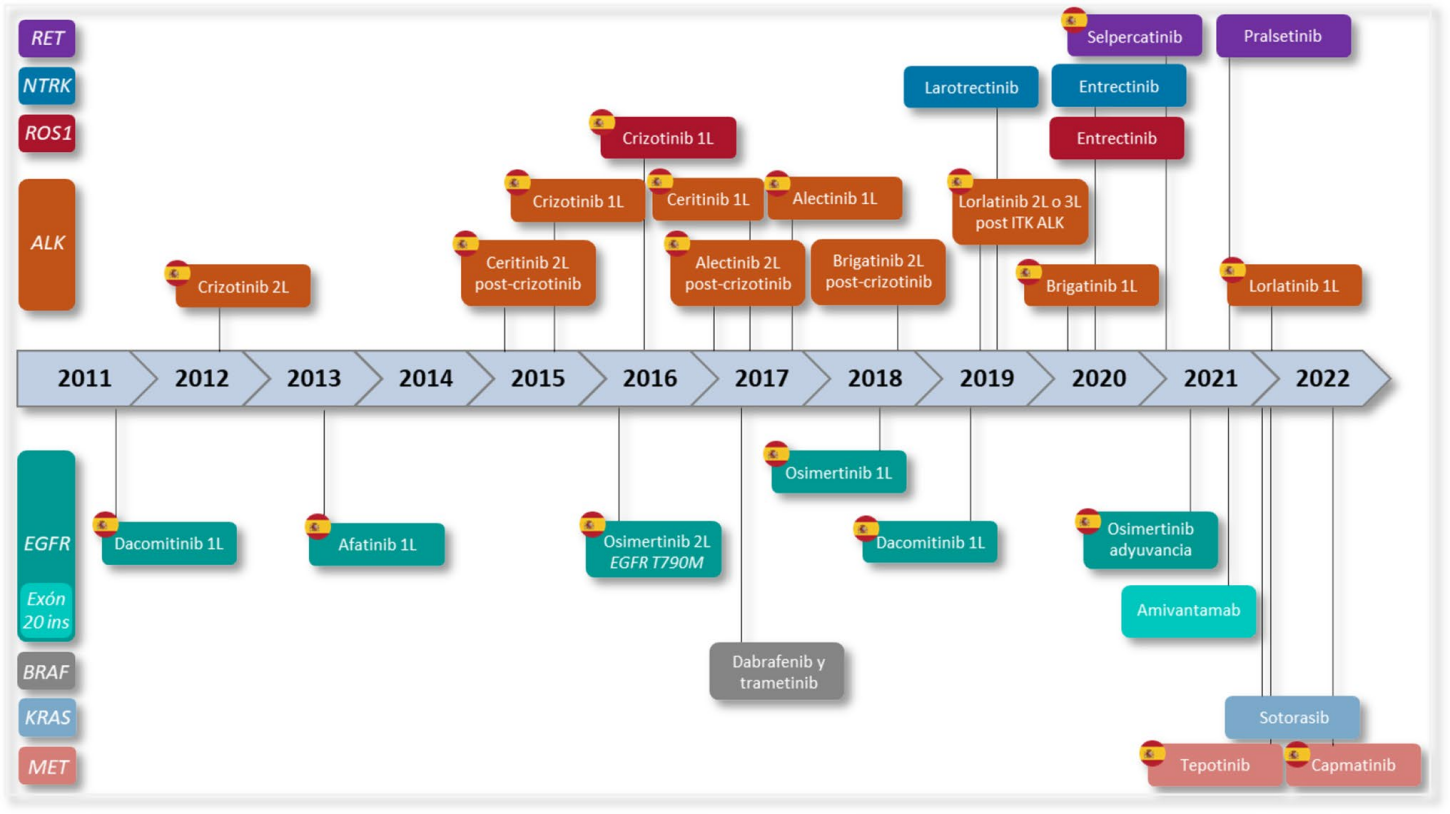
Adapted from [4]. Timeline of EMA-approved targeted therapies for non-small cell lung cancer with actionable alterations. Drug availability in Spain is represented by the flag icon

However, the availability of these treatments in Spain is not complete due to the lack of reimbursement for innovative treatments or delayed funding, only five of these driver alterations are currently covered from a therapeutic point of view (Fig. 1) [6].

In this paper, we analyzed the barriers delaying the incorporation of these innovative treatments into the Spanish National Health System (SNS).

## Materials and methods

A literature review was conducted of all relevant content published on the websites of the European Commission (EC), the European Medicines Agency (EMA), and the Spanish Agency of Medicines and Medical Products (AEMPS) regarding the authorization of and access to oncology treatments.

## Results

### Evidence on barriers to access to innovative treatments

Access to cancer care innovations will be an important metric to measure whether cancer patients have access to clinical advances.

**Fig. 2:**
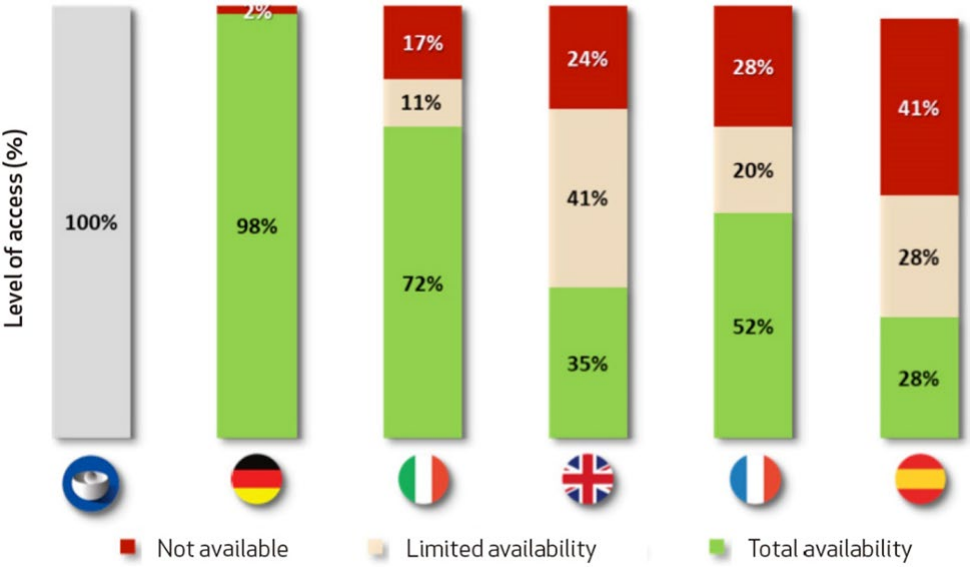
Adapted from the report “EFPIA Patients W.A.I.T. Indicator 2022 Survey”. Comparison of the state of funding of oncology drugs approved by the European Medicines Agency between Germany, Italy, the United Kingdom, France, and Spain between 2018 and 2021

Several reports have been published to demonstrate the level of uptake of innovation in oncology. One of the most relevant reports in terms of its history (more than 15 years) is the “EFPIA Patients W.A.I.T. Indicator Survey” [7], published bi-annually since 2004 by the European Federation of Pharmaceutical Industries and Associations (EFPIA). This document analyzes the funding situation in several European countries, allowing a comparative analysis. The access indicators analyzed are the percentage of treatment availability and the time to funding. Regarding the percentage of availability of oncology drugs, it can be observed that, compared to other European countries such as Germany, Italy, United Kingdom, and France, Spain is the country with the lowest level of access to cancer drugs approved by the EMA. Only 28% of the drugs approved by the EMA between 2018 and 2021 are fully available to patients, while 28% are available with restrictions and 41% are unavailable (Fig. 2).

**Fig. 3:**
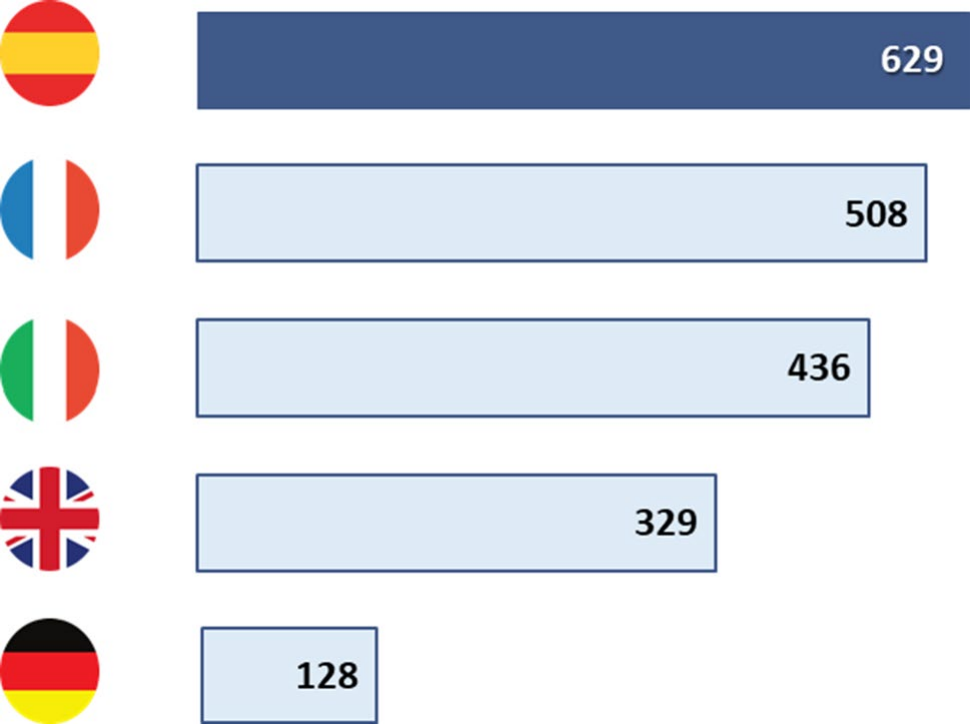
Adapted from the report “EFPIA Patients W.A.I.T. Indicator 2022 Survey”. Comparison of the average time to financing of drugs since their approval by the EMA (2018–2021) between Spain, France, Italy, the United Kingdom, and Germany

In terms of the time taken to adopt a pharmaceutical innovation, the average in Spain is 629 days for all medicines and 611 days for a cancer drug, making it the country with the longest time from EMA approval to adoption of the innovation among the countries mentioned above (Fig. 3). This time has worsened over the years and is now more than 100 days longer than in the previous report (629 vs 517 days).

Societies and scientific groups, such as the Spanish Lung Cancer Group (SLCG) or the ECO Foundation, have expressed their concern about access to oncological innovation in our country by publishing reports analyzing information from the W.A.I.T. published in 2022. The SLCG, in 2021, published the report “Delays and restrictions in access to innovative treatments for lung cancer in Spain” with the aim of “estimating the potential loss of clinical benefit implied by delays in the incorporation of innovation” [8]. In both reports, the experts concluded that the situation in Spain regarding the availability of innovation has worsens in recent years. This has a direct impact on patients, who are deprived of the potential clinical benefits of medicines. The ECO Foundation recently launched the ECO Observatory, an initiative designed to highlight the importance of patient access to oncology innovation. The results are included in a report recently published in collaboration with IQVIA. This report analyzes the current situation in Spain and identifies possible ways to improve patient access to oncological innovation [9]. The findings from the ECO Observatory are consistent with those of the recent W.A.I.T. report: between 01/01/2018 and 06/30/2021, only 38% EMA-approved drugs and 36% of new indications were included in supply on 01/12/2022.

Furthermore, in terms of the time to complete the pricing and financing process, it took on average 16.2 months to include new medicines and 17.8 months to finance new oncological indications [10].

The Ministry of Health has also produced a report on the ‘Evolution of the financing and pricing of oncological drugs in the SNS (2016–2021)’ which shows similar results to those previously described by both EFPIA and the ECO Observatory in terms of the time taken to finance cancer drugs, which is always more than 1 year (416 days).

The Spanish Society of Medical Oncology (SEOM) and the Spanish Society of Hematology and Hemotherapy (SEHH) have submitted a document to the AEMPS containing 12 aims to improve 4 aspects of the process: registration procedures, positioning reports, assessment nodes, and pricing and funding. These included ensuring the transparency and traceability of the registration process for new drug or indication, specifying the duration of the pilot phase of the consolidation plan for Therapeutic Positioning Reports (IPTs) and publishing the criteria for funding medicines or indications.

**Fig. 4:**
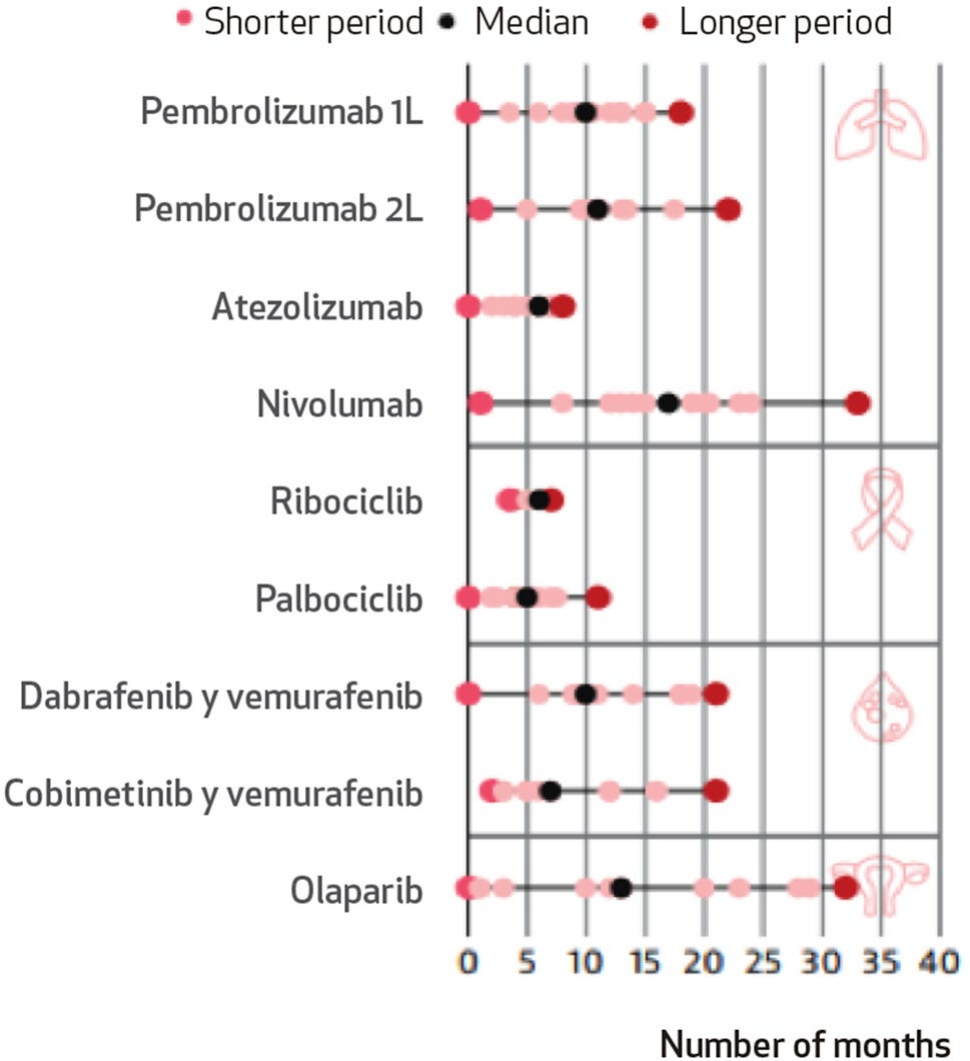
Time to access innovative cancer treatments in the different Autonomous Communities. Source: National Cancer Profiles 2023 Report (Spain), published by the Organization for Economic Co-operation and Development (OECD). Note: the data refer to the 17 Autonomous Communities and the Autonomous City of Ceuta. They were collected from 58% of the centers contacted in the first quarter of 2019 and include relevant drugs with pricing and reimbursement conditions established between January 2016 and April 2018 for lung, breast, melanoma, and uterine cancer

It is important to remember that Spain has a decentralized health system. This means that for a medicine to be used in the health centers of the SNS, it must not only be included in the portfolio of common services of the SNS, but it must also be available regionally in each of the Autonomous Communities (AACC), which cover the cost of the medicine (Fig. 4).

To all these reports, we need to add the perspective of patients, who also express their concern about the lack of access to oncological innovation in Spain, as they are the most affected by this situation. In the specific case of lung cancer, the Spanish Association of People Affected by Lung Cancer (AEACaP) has drawn up a document in which it asks the health authorities for “rapid and equitable access to medicines for lung cancer” given the rejection and lack of funding for various medicines by the Ministry of Health [11].

### Analysis of the situation of access to innovative treatments for lung cancer in Spain

**Fig. 5:**
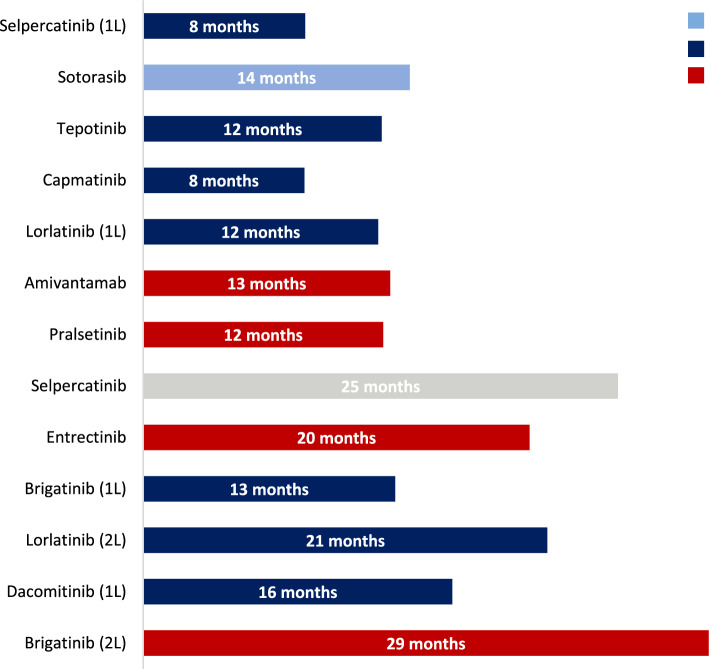
Time (in months) from European approval to inclusion in the SNS (dark blue), exclusion from reimbursement (red) or indication/treatment still under investigation (light blue). Selpercatinib is shown in grey because this indication was replaced by the first-line indication in the marketing authorization and therefore no longer appears as such

We reviewed the status of new treatments/indications approved by the EMA since 2018 (Table 1, Fig. 5).

**Table 1:** New treatments approved by the EMA since 2018

Drug	Mechanism of action	Pharmaceutical laboratory	Indication	EMA approval	Pivotal clinical trial	Status	Potential delay
1. Brigatinib	ALK inhibitor	Takeda	1.1. Monotherapy for adult patients with advanced, anaplastic lymphoma kinase (ALK)-positive NSCLC who have been previously treated with crizotinib	November 2018	Phase I/II	Not funded by resolution **May 2021**	≥ 29 months
			1.2. Monotherapy for adult patients with advanced, anaplastic lymphoma kinase (ALK)-positive NSCLC who have not been previously treated with an ALK inhibitor	April 2020	Phase III	Indication funded **May 2021**	13 months
2. Dacominitib	EGFR inhibitor	Pfizer	2.1. Monotherapy for adult patients with locally advanced or metastatic advanced NSCLC with epidermal growth factor receptor (EGFR) activating mutations	April 2019	Phase III	Indication funded **August 2020**	16 months
3. Lorlatinib	ALK/ROS1 inhibitor	Pfizer	3.1. Monotherapy for advanced ALK + NSCLC after progression to (i) alectinib or ceritinib as a first treatment with an ALK inhibitor, or (ii) crizotinib and at least one other ALK inhibitor	May 2019	Phase II	Indication funded **February 2021**	21 months
			3.2. Monotherapy for adult patients with advanced anaplastic lymphoma kinase (ALK)-positive NSCLC not previously treated with an ALK inhibitor	January 2022	Phase III	Indication funded **February 2023**	12 months
4. Larotrectinib	NTRK inhibitor	Bayer	4.1. Monotherapy for the treatment of adult and pediatric patients with solid tumors with NTRK fusion, locally advanced, metastatic or whose surgical resection is likely to result in high morbidity, and in the absence of satisfactory therapeutic options	September 2019	Phase I/II	Not funded by resolution **April 2022**	≥ 31 months
5. Entrectinib	ROS1/NTRK inhibitor	Roche	5.1. Monotherapy for adult patients with advanced ROS1-positive NSCLC not previously treated with ROS1 inhibitors	July 2020	Phase I/II	Not funded by resolution **April 2022**	≥ 20 months
			5.2. Monotherapy for adult and pediatric patients 12 years of age and older, with solid tumors expressing a neurotrophic tyrosine kinase receptor (NTRK) gene fusion, who have not previously received an NTRK inhibitor				
6. Selpercatinib	RET inhibitor	Lilly	6.1. Monotherapy for adults with advanced NSCLC, RET gene fusion positive, not previously treated with a RET inhibitor^a^	June 2022 *February 2021	Phase I/II	Indication funded	8 months *26 months
7. Pralsetinib	RET inhibitor	Roche	7.1. Monotherapy for adult patients with RET gene fusion-positive advanced NSCLC not previously treated with a RET inhibitor	November 2021	Phase I/II	Not funded by resolution **December 2022**	≥ 12 months
8. Amivantamab	EGFR and MET inhibitor	Janssen	8.1. Monotherapy for adult patients with advanced NSCLC with activating insertional mutations in exon 20 of the epidermal growth factor receptor (EGFR), after failure of platinum-based therapy	December 2021	Phase I/II	Not funded by resolution **March 2023**	≥ 13 months
9. Capmatinib	MET inhibitor	Novartis	9.1. Monotherapy for adult patients with advanced NSCLC who have alterations resulting in a skipping of exon 14 of the mesenchymal–epithelial transition factor gene (METex14), requiring systemic treatment after prior treatment with immunotherapy and/or platinum-based chemotherapy	June 2022	Phase II	Indication funded **March 2023**	8 months
10. Tepotinib	MET inhibitor	Merck Serono	10.1. Monotherapy for adult patients with advanced NSCLC with exon 14 gene skipping alterations (METex14), requiring systemic treatment after prior treatment with immunotherapy and/or platinum-based chemotherapy	February 2022	Phase II	Indication funded **March 2023**	12 months
11. Sotorasib	KRAS G12C inhibitor	Amgen	11.1. Monotherapy for adults with advanced NSCLC with KRAS G12C mutation and who have progressed after at least one line of previous systemic treatment	January 2022	Phase I/II	Under study. First refusal in February 2023	≥ 14 months
12. Dabrafenib–trametinib	BRAF and MEK inhibitors	Novartis	12.1. Dabrafenib in combination with trametinib is indicated for the treatment of adult patient with advanced NSCLC with BRAF V600 mutation	February 2017	Phase II	Not funded by resolution **January 2018**	11 months

^a^The EMA initially approved selpercatinib for patients with advanced, RET gene fusion-positive non-small-cell lung cancer (NSCLC) requiring systemic treatment after prior treatment with immunotherapy and/or platinum-based chemotherapy

#### I. Brigatinib (Alunbrig^®^) [12–21]

1.1. Brigatinib indications funded by SNS in Spain, according to EMA Data Sheet:
  SNS indication #1: Brigatinib monotherapy is indicated for the treatment of adult patients with advanced *ALK*-positive NSCLC who have not been previously treated with an ALK tyrosine kinase inhibitor (TKI) [12]:
    Clinical evidence approval SNS indication #1: Phase III randomized study ALTA-1L (NCT02737501) evaluating efficacy and safety of brigatinib in patients with *ALK*-positive NSCLC who had not received prior treatment with an ALK TKI. Progression-free survival (PFS) was significantly higher in those patients who received brigatinib versus those who were treated with crizotinib [13, 14].
    EC indication approval date: April 2020 [15].
    Delay between EC approval and funding in Spain: Funding in May 2021 (397 days) [16].
    Number of times in Inter-Ministerial Commission for the Pricing of Medicines (CIPM): 1, CIPM 210 [17].
1.2. Brigatinib indications published in EMA Data Sheet not funded by SNS in Spain:
  EMA indication #2: Brigatinib monotherapy is indicated for treatment of adult patients with advanced *ALK*-positive NSCLC previously treated with crizotinib [12]:
    Clinical evidence approval EMA indication #2: Phase II, open-label, randomized study, ALTA (NCT02094573), which evaluated efficacy and safety of brigatinib in patients with *ALK*-positive NSCLC refractory to crizotinib [18, 19].
    EMA #2 indication approval date: November 2018 [20].
    Delay between EC approval and funding in Spain: Funding rejected by decision in May 2021 (≥ 891 days) [16].
    Number of times in CIPM: 2, CIPM 208, CIPM 210. Reason for non-funding (CIPM 210): “high uncertainty about its clinical benefits and the existence of other therapeutic alternatives with better clinical evidence and lower treatment costs” [17, 21].

#### II. Dacomitinib (Vizimpro^®^) [22–27]

2.1. Dacomitinib indications funded by SNS in Spain, according to EMA Data Sheet:
  SNS indication #1: Dacomitinib monotherapy is indicated for first-line treatment of adult patients with locally advanced or metastatic NSCLC with activating *EGFR* mutations [22].
    Clinical evidence approval SNS indication #1: Phase III ARCHER 1050 study, active treatment controlled, evaluating efficacy and safety in previously untreated patients with *EGFR* mutations [23].
    EC indication approval date: April 2019 [24].
    Delay between EC approval and funding in Spain: Financed by decision in August 2020 (487 days) [25].
    Number of times in CIPM: 2, CIPM 198 and CIPM 200. In the first CIPM, it was agreed not to include dacomitinib for reasons “of rationalization of public expenditure on pharmaceutical services, taking into account its unfavorable cost-effectiveness ratio compared to the alternative to the pivotal trial” [26, 27].
2.2. Dacomitinib indications published in EMA Data Sheet not funded by SNS in Spain:
  N/A

#### III. Lorlatinib (Lorviqua®) [21, 28–35]

3.1. Lorlatinib indications funded by SNS in Spain, according to EMA Data Sheet:
  SNS indication #1: Lorlatinib monotherapy is indicated for treatment of adult patients with *ALK*-positive advanced NSCLC whose disease has progressed after alectinib or ceritinib as the first ALK TKI therapy or crizotinib and at least one other ALK TKI [28].
    Clinical evidence approval SNS indication #1: Phase I/II study B7461001, non-randomized, open, multicohort and without comparator, which evaluated efficacy and safety of lorlatinib in patients with *ALK-*positive or *ROS1*-positive advanced NSCLC [29].
    EC indication approval date: May 2019 [30].
    Delay between EC approval and funding in Spain: Financed by decision in February 2021 (637 days) [31].
    Number of times in CIPM: 1, CIPM 208 [21].
  SNS indication #2: Lorlatinib monotherapy is indicated for adult patients with *ALK*-positive advanced NSCLC not previously treated with an ALK TKI [28].
    Clinical evidence approval SNS indication #2: Phase III B7461006 CROWN study, a multicenter, randomized (1:1), open-label, controlled trial with active treatment (crizotinib), evaluating efficacy of lorlatinib in patients who had not received prior treatment. Lorlatinib may be considered a suitable treatment option for *ALK*+ advanced NSCLC patients who have not received prior ALK TKI treatment [32].
    EC indication approval date: January 2022 [33].
    Delay between EC approval and funding in Spain: Financed by decision in February 2023 (370 days) [31].
    Number of times in CIPM: 2, CIPM 227 and CIPM 230. The CIPM proposed not to fund the drug based on “criteria for rationalizing public expenditure on pharmaceutical services and the budgetary impact on the National Health System. The Commission has considered the financial uncertainty of this indication (a very long duration of treatment is estimated) and the existence of other therapeutic alternatives with more evidence.” [34, 35].
3.2. Lorlatinib indications published in EMA Data Sheet not funded by SNS in Spain:
  N/A

#### IV. Larotrectinib (Vitrakvi^®^) [36–42]

4.1. Larotrectinib indications funded by SNS in Spain, according to EMA Data Sheet:
  N/A
4.2. Larotrectinib indications published in EMA Data Sheet not funded by SNS in Spain:
  EMA indication #1: Larotrectinib monotherapy is indicated for treatment of adult and pediatric patients with solid tumors that harbor a *NTRK* gene fusion, whose disease is locally advanced, metastatic, or where surgical resection is likely to result in severe morbidity, and who have no satisfactory treatment options [36].
    Clinical evidence approval EMA indication #1: Several studies, including the phase I study LOXO-TRK-14001 (NCT02122913), in adult patients, the phase II study NAVIGATE (NCT02576431), in adult patients and adolescents, and the phase I/II SCOUT study (NCT02637687) in pediatric patients. Two of these studies, NCT02122913 and NCT02576431, confirmed the activity and safety of larotrectinib in patients with advanced-stage *NTRK* gene fusion lung cancer, including those with central nervous system (CNS) metastases. A total of 14 patients (13 NSCLC and 1 SCLC) were analyzed. Of these, seven patients (six NSCLC and one SCLC) had metastases in the CNS at baseline [37–39].
    EC indication approval date: September 2019 [40].
    Delay between EC approval and funding in Spain: Funding rejected by decision in April 2022 (≥ 643 days) [41].
    Number of times in CIPM: 2, CIPM 212, CIPM 221. Reason for non-funding (CIPM 221): “proposal submitted by the company which does not resolve the uncertainties regarding its therapeutic value. In addition, the criteria for rationalizing of public expenditure and the budgetary impact of the SNS have been considered.” [42].

#### V. Entretinib (Rozlytrek^®^) [35, 43–48]

5.1. Entrectinib indications funded by SNS in Spain, according to EMA Data Sheet:
  N/A
5.2. Entrectinib indications published in EMA Data Sheet not funded by SNS in Spain:
  EMA indication #1: Entrectinib monotherapy is indicated for treatment of adult and pediatric patients 12 years of age and older, with solid tumors *NTRK* gene fusion, whose disease is locally advanced, metastatic, or where surgical resection is likely to result in severe morbidity, and who have not received a prior *NTRK* inhibitor and who have no satisfactory treatment options [43].
    Clinical evidence approval EMA indication #1: Several studies, including the phase I ALKA study (NCT02097810), phase I STARTRK-1 study (NCT02097810), and phase II STRATRK-2 study (NCT02568267), all of them in adult patients including patients with molecular alterations of *NTRK*1/2/3, *ROS1*, and *ALK*. The pooled analysis included 93 patients with *NTRK* fusion-positive, 53 *ROS1*-positive NSCLC patients from these 3 trials, which was expanded to 94 patients as requested by the CHMP. All patients were diagnosed at an advanced stage and patients with CNS metastases were included. After a median follow-up of 23.9 months, the data reported with entrectinib are considered positive in tumors with few therapeutic options or with alternatives of limited efficacy [44].
    EC indication approval date: July 2020 [45].
    Delay between EC approval and funding in Spain: Not funded by resolution in April 2022 (≥ 609 days) [46].
    Number of times in CIPM: 4, CIPM 213, CIPM 221, CIPM 230, and CIPM 232. The CIPM proposed non to fund the SNS due to “uncertainties regarding its therapeutic value and rationalization criteria for public expenditure and the budgetary impact of the SNS.” [35, 42, 47].
  EMA indication #2: Entrectinib monotherapy is indicated for treatment of adult patients with *ROS1-*positive advanced NSCLC not previously treated with ROS1 inhibitors [43].
    Clinical evidence approval EMA indication #2: Several studies, including the phase I ALKA study (NCT02097810), phase I STARTRK-1 study (NCT02097810), and phase II STRATRK-2 study (NCT02568267), all of them in adult patients. All 53 *ROS1*-positive NSCLC patients from these 3 trials were included in the pooled analysis, which was expanded to 94 patients as requested by the CHMP. All patients were diagnosed at an advanced stage and patients with CNS metastases were included. After a median follow-up of 20.3 months, entrectinib has demonstrated activity in the treatment of ROS1-inhibitor-naïve patients with *ROS1*-positive advanced NSCLC [48].
    EC indication approval date: July 2020 [45].
    Delay between EC approval and funding in Spain: Non-funding resolution April 2022 (≥ 609 days) [46].
    Number of times in CIPM: 4, CIPM 213, CIPM 221, CIPM 230, and CIPM 232. Reason for non-funding: “uncertainties regarding its therapeutic value and the criteria for rationalizing public expenditure and the budgetary impact of the SNS.” [35, 42, 47].

#### VI. Selpercatinib (Retsevmo^®^) [49–54]

6.1. Selpercatinib indications funded by SNS in Spain, according to EMA Data Sheet:
  SNS indication #1: Selpercatinib monotherapy is indicated for treatment of patients with *RET* fusion-positive advanced NSCLC not previously treated with a RET inhibitor. Although this is the indication that currently appears on the Data Sheet, selpercatinib was originally approved by the EC for patients who had previously been treated with immunotherapy and/or platinum-based chemotherapy [49].
    Clinical evidence approval SNS indication #1: The approval of both indications is based on the results of the phase I/II LIBRETTO-001 (NCT03157128) study, which evaluated selpercatinib, separately, in patients with advanced NSCLC with *RET* oncogene fusion, treated and not previously treated with platinum-based chemotherapy. In this study, patients treated with selpercatinib achieved high response rates and long-lasting clinical benefit, all associated with a favorable safety profile. Selpercatinib is the first therapy specifically indicated for the treatment of adult patients with *RET* oncogene fusion metastatic NSCLC [50].
    EC indication approval date: February 2021 [51].
    Date amended by the EC: September 2022 [52].
    Delay between EC approval and funding in Spain: Financed by resolution (≥ 748 days and 254 days) [53].
    Number of times in CIPM: 2, CIPM 225 and CIPM 232. The first CIPM rejected the application because “the currently available data (preliminary data from a single-arm, open-label, phase I/II trial with objective response rate as the primary efficacy variable) and the company's financing proposal do not resolve the economic and financial uncertainties of the drug” [54]. Following the allegations in CIPM 232, the Commission agreed to propose to the AEMPS to accept the allegations and include this medicine.
6.2. Selpercatinib indications published in EMA Data Sheet not funded by SNS in Spain:
  N/A

#### VII. Pralsetinib (Gavreto^®^) [55–59]

7.1. Pralsetinib indications funded by SNS in Spain, according to EMA Data Sheet:
  N/A
7.2. Pralsetinib indications published in EMA Data Sheet not funded by SNS in Spain:
  EMA indication #1: Pralsetinib monotherapy is indicated for treatment of adult patients with *RET* fusion-positive advanced NSCLC not previously treated with a RET inhibitor [55].
    Clinical evidence approval EMA indication #1: Phase I/II ARROW, multicenter, non-randomized, open, non-controlled, multicohort clinical trial. In phase II, the clinical efficacy of pralsetinib was evaluated in nine cohorts in various tumors with RET activity, mainly NSCLC and thyroid cancer. Specifically in NSCLC, pralsetinib produced high antitumor responses and brain activity in patients pre-treated with platinum-based chemotherapy with or without immunotherapy [56].
    EC indication approval date: November 2021 [57].
    Delay between EC approval and funding in Spain: Not funded by resolution in December 2022 (≥ 378 days) [58].
    Number of times in CIPM: 2, CIPM 225 and CIPM 227. Reason for not funding: “the currently available data (preliminary data from a single-arm, open-label, phase I/II trial with objective response rate as a variable principle of efficacy) and the company's funding proposal do not address the economic and financial uncertainties of the drug” [54, 59].

#### VIII. Amivantamab (Rybrevant^®^) [60–65]

8.1. Amivantamab indications funded by SNS in Spain, according to EMA Data Sheet:
  N/A
8.2. Amivantamab indications published in EMA Data Sheet not funded by SNS in Spain:
  EMA indication #1: Amivantamab monotherapy is indicated for treatment of adult patients with advanced NSCLC with activating *EGFR* exon 20 insertion mutations, after failure of a platinum-based therapy [60].
    Clinical evidence approval EMA indication #1: Phase I/II CHRYSALIS, multicenter, open label, multicohort study conducted to assess the safety and efficacy of amivantamab in patients with locally advanced or metastatic NSCLC. The approved population is patients with locally advanced or metastatic NSCLC harboring *EGFR* exon 20 insertion mutations whose disease has progressed on or after platinum-based chemotherapy. In this study, patients treated with amivantamab achieved high response rates and durable clinical benefit, all with a favorable safety profile. Amivantamab is the first therapy specifically indicated for the treatment of adult patients with metastatic NSCLC harboring *EGFR* exon 20 insertion mutations [61].
    EC indication approval date: December 2021 [62].
    Delay between EC approval and funding in Spain: Not funded by resolution in March 2023 (≥ 389 days) [63].
    Number of times in CIPM: 2, CIPM 229 and CIPM 231. Reason for non-financing (CIPM 231): "consideration of criteria for rationalizing public expenditure and the budgetary impact of the SNS" [64, 65].

#### IX. Capmatinib (Tabrecta^®^) [66–69]

9.1. Capmatinib indications funded by SNS in Spain, according to EMA Data Sheet:
  SNS indication #1: Capmatinib monotherapy is indicated for treatment of adult patients with advanced NSCLC who have *MET*ex14 skipping mutations and who require systemic treatment after prior treatment with immunotherapy and/or platinum-based chemotherapy [66].
    Clinical evidence approval SNS indication #1: Approval based on the results of the phase II GEOMETRY mono-1, non-randomized, multicohort study in patients with NSCLC with different MET alterations (amplifications or mutations) and different lines of treatment (naïve or second /third lines). For the second and subsequent lines of treatment, capmatinib showed antitumor activity (high number of relatively durable responses) that can be considered sufficient [67].
    EC indication approval date: June 2022 [68].
    Delay between EC approval and funding in Spain: Financed by resolution in March 2023 (254 days) [69].
    Number of times in CIPM: 2, CIPM 229 and CIPM 231. The first CIPM agreed to propose non-funding on the grounds that "the clinical uncertainties arising from the trial are not resolved by the proposal submitted by the company, which also represents a very high cost of treatment. [64, 65].
9.2. Capmatinib indications published in EMA Data Sheet not funded by SNS in Spain:
  N/A

#### X. Tepotinib (Tepmetko^®^) [69–72]

10.1. Tepotinib indications funded by SNS in Spain, according to EMA Data Sheet:
  SNS indication #1: Tepotinib monotherapy is indicated for treatment of adult patients with advanced NSCLC harboring *MET*ex14 skipping mutations, who require systemic treatment after prior treatment with immunotherapy and/or platinum-based chemotherapy [70].
    Clinical evidence approval SNS indication #1: phase II, multicenter, open-label, single-arm, VISION study in patients with advanced NSCLC *MET*ex14 skipping mutations. In this study, the use of tepotinib was associated with partial responses in almost half of the patients [71].
    EC indication approval date: February 2022 [72].
    Delay between EC approval and funding in Spain: financed by resolution in March 2023 (376 days) [69].
    Number of times in CIPM: CIPM 229 and CIPM 231. The first CIPM was agreed to propose non-funding on the grounds of “uncertainty of therapeutic value and rationalization criteria for public expenditure on pharmaceutical services and budgetary impact.” [64, 65].
10.2. Tepotinib indications published in EMA Data Sheet not funded by SNS in Spain:
  N/A

#### XI. Sotorasib (Lumykras^®^) [73–76]

11.1. Sotorasib indications funded by SNS in Spain, according to EMA Data Sheet:
  N/A
11.2. Sotorasib indications published in EMA Data Sheet not funded by SNS in Spain:
  EMA #1 indication: Sotorasib monotherapy is indicated for treatment of adults with advanced NSCLC with *KRAS* G12C mutation and who have progressed after at least one prior line of systemic therapy [73].
    Clinical evidence approval EMA indication #1: Phase I/II CodeBreak100 (NCT03600883) clinical trial in patients with advanced NSCLC *KRAS* G12C mutation who had progressed after at least one prior treatment. Sotorasib is the first drug approved in Europe for patients with advanced *KRAS* G12C mutated NSCLC [74].
    EC indication approval date: January 2022 [75].
    Delay between EC approval and funding in Spain: Non-financing proposal in CIPM 231 (≥ 420 days) [76].
    Number of times in CIPM: 1, CIPM 231. Reason for proposal not to finance (CIPM 231): "taking into account the criteria for rationalizing public expenditure and the budgetary impact of the SNS." [63].

#### XII. Dabrafenib (Tafinlar^®^)—trametinib (Mekinist^®^)

12.1. Dabrafenib–trametinib indications funded by SNS in Spain, according to EMA Data Sheet:N/A
  N/A
12.2. Dabrafenib–trametinib indications published in EMA Data Sheet not funded by SNS in Spain:
  EMA #1 indication: Dabrafenib in combination with trametinib is indicated for treatment of adult patient with advanced NSCLC with BRAF V600 mutation [77, 78].
    Clinical evidence approval EMA indication #1: The efficacy and safety were studied in a phase II, BRF113928, three-cohort study, multicenter, non-randomized and open-label study. Ninety-three patients with stage IV BRAF V600E mutant NSCLC were enrolled in the combination therapy cohorts. ORR in the first line population was 61.1% and in the previously treated population was 66.7%.
    EC indication approval date: February 2017 [79].
    Delay between EC approval and funding in Spain: Non-financing proposal in CIPM 179 (≥ 330 days) [80].
    Number of times in CIPM: 1, CIPM 179. Reason for proposal not to finance (CIMP 179): “taking into account the criteria for rationalizing public expenditure and the budgetary impact of the SNS” [81].

## Discussion

In the last decade, more than 15 drugs covering more than 20 indications have been approved by the EC to treat patients with lung cancer harboring oncogenic driver molecular alterations. Fifteen drugs were approved between 2018 and 2023. We observed a common denominator when reviewing the latest EMA approvals:

1. Except for capmatinib, which was reimbursed in 8 months after EC approval, all EMA-approved indications or innovative medicines took more than a year to be funded, with an average of approximately 14 months from EC approval to reimbursement.
2. The average number of times that a drug/indication is presented to the CIPM is more than once. Apart from lorlatinib, no other drug received a funding agreement at the first CIPM.
3. Before 2023, only lorlatinib achieved funding with a single-arm phase I/II study in its second-line indication. A review of the reasons for non-inclusion in the SNS pathway shows that clinical uncertainty is the most cited reason. Randomized clinical trials to demonstrate the superiority over standard of care is not feasible and probably not strictly needed in the context of highly selected population based on a driver alteration that is very uncommon in the general population.
4. Advances in precision oncology are not yet reality in Spain. For patients with *EGFR* exon 20 insertion, *NTRK* fusion, *KRAS* G12C, and *BRAF* V600E mutations, no specific treatments are available due to lack of funding. These mutations are low frequency, but collectively they account for about 20% of patients diagnosed with a lung adenocarcinoma, which represent a large population denied access to innovation.
5. Lack of availability of effective targeted therapy for lung cancer patients with actionable genomic alterations is concerning, because it denies patients potential clinical benefit, both in terms of overall survival and quality of life.

## Conclusion

There is, therefore, a significant inequity in terms of access to cancer treatments across distinct European countries. Unfortunately, Spain is one of the countries with the longest average time from EMA approval to drug reimbursement. In this article, we focused on treatments indicated on patients with NSCLC with a driver alteration in which it was shown that the barriers to precision medicine have major impact on their life expectancy and quality of life. The SLCG undertakes to report this inequity and to strive to improve the situation of access to oncological medicines in our country.

## Data Availability

Not applicable.

## References

[1] Siegel RL, Miller KD, Sandeep Wagle N, Jemal A (2023). Cancer statistics, 2023. CA Cancer J Clin.

[2] Las cifras del cáncer en España 2023. Depósito Legal: M-3407–2023. ISBN: 978-84-09-48173-6. 2023. Sociedad Española de Oncología Médica (SEOM).

[3] Goldstraw P, Chansky K, Crowley J, Rami-Porta R, Asamura H, Eberhardt WEE (2016). The IASLC Lung Cancer Staging Project: proposals for revision of the TNM stage groupings in the forthcoming (eighth) edition of the TNM classification for lung cancer. J Thorac Oncol.

[4] Tan AC, Tan DSW (2022). Targeted therapies for lung cancer patients with oncogenic driver molecular alterations. J Clin Oncol.

[5] European Commission. Public Health – Union Register of medicinal products: https://ec.europa.eu/health/documents/community-register/html/reg_hum_act.htm?sort=n.

[6] BIFIMED: Buscador de la Información sobre la situación de financiación de los medicamentos: https://www.sanidad.gob.es/profesionales/medicamentos.do?metodo=buscarMedicamentos.

[7] European Federation of Pharmaceutical Industries and Associations (EFPIA). EFPIA Patients W.A.I.T. Indicator 2022 Survey. https://www.farmaindustria.es/web/wp-content/uploads/sites/2/2023/04/EFPIA-Patient-W.A.I.T.-Indicator-Final-report.pdf.

[8] Informe del GECP “Demoras y restricciones en el acceso a tratamiento innovadores para el cáncer de pulmón en España”. Rev Esp Econ Salud 16:2. Disponible en: https://www.gecp.org/wp-content/uploads/2021/05/informe_gecp.pdf.

[9] Informe Observatorio ECO “Reflexión sobre la situación actual de España y posibles áreas de mejora en el acceso de los pacientes a la innovación oncológica”. Disponible en: https://gepac.es/cuestionestado/pdf/Informe%20Observatorio%20Eco.pdf

[10] Documento Ministerio de Sanidad

[11] Manifiesto por un acceso ágil y equitativo a fármacos para cáncer de pulmón. https://afectadoscancerdepulmon.com/manifiesto-por-un-acceso-agil-y-equitativo-a-farmacos-para-cancer-de-pulmon

[12] Ficha técnica Alunbrig (brigatinib). https://cima.aemps.es/cima/dochtml/ft/1181264011/FT_1181264011.html

[13] Camidge DR, Kim HR, Ahn M-J, Yang JC-H, Han J-Y, Lee J-S (2018). Brigatinib versus crizotinib in ALK-positive non-small-cell lung cancer. N Engl J Med.

[14] Camidge DR, Kim HR, Ahn M-J, Yang JC-H, Han J-Y, Hochmair MJ (2021). Brigatinib versus crizotinib in ALK inhibitor-naïve advanced ALK-positive NSCLC: final results of phase 3 ALTA-1L trial. J Thorac Oncol.

[15] Decisión de ejecución de la Comisión de 1.4.2020. https://ec.europa.eu/health/documents/community-register/2020/20200401147675/dec_147675_es.pdf.

[16] https://www.sanidad.gob.es/profesionales/medicamentos.do?metodo=verDetalle&cn=726758.

[17] Acuerdos de la reunión de la Comisión Interministerial de precios de los medicamentos. https://www.sanidad.gob.es/profesionales/farmacia/pdf/ACUERDOS_CIPM_210_3_marzo_2021_web.pdf.

[18] Kim DW, Tiseo M, Ahn M-J, Reckamp KL, Hasen KH, Kim S-W (2017). Brigatinib in patients with crizotinib-refractory anaplastic lymphoma kinase-positive non-small-cell lung cancer: a randomized, multicenter phase II trial. J Clin Oncol.

[19] Huber RM, Hansen KH, Paz-Ares Rodríguez L, West HL, Reckamp KL, Leighl NB (2020). Brigatinib in crizotinib-refractory ALK+ NSCLC: 2-year follow-up on systemic and intracranial outcomes in the phase 2 ALTA trial. J Thorac Oncol.

[20] Decisión de ejecución de la Comisión de 22.11.2018. https://ec.europa.eu/health/documents/community-register/2018/20181122142707/dec_142707_es.pdf.

[21] Acuerdos de la reunión de la Comisión Interministerial de precios de los medicamentos. https://www.sanidad.gob.es/profesionales/farmacia/pdf/Acuerdos_CIPM_208_de_17_de_DICIEMBRE_web.pdf.

[22] Ficha técnica Vizimpro (dacomitinib). https://cima.aemps.es/cima/dochtml/ft/1191354001/FT_1191354001.html.

[23] Wu Y-L, Cheng Y, Zhou X, Lee KH, Nakagawa K, Niho S (2017). Dacomitinib versus gefitinib as first-line treatment for patients with EGFR-mutation-positive non-small-cell lung cancer (ARCHER 1050): a randomized, open-label, phase 3 trial. Lancet Oncol.

[24] Decisión de ejecución de la Comisión de 2.4.2019. https://ec.europa.eu/health/documents/community-register/2019/20190402143982/dec_143982_es.pdf.

[25] https://www.sanidad.gob.es/profesionales/medicamentos.do?metodo=verDetalle&cn=725347.

[26] Acuerdos de la reunión de la Comisión Interministerial de precios de los medicamentos. https://www.sanidad.gob.es/profesionales/farmacia/pdf/ACUERDOS_DE_LA_CIPM_198_web.pdf.

[27] Acuerdos de la reunión de la Comisión Interministerial de precios de los medicamentos. https://www.sanidad.gob.es/profesionales/farmacia/pdf/ACUERDOS_DE_LA_CIPM_200_web.pdf.

[28] Ficha técnica Lorviqua (lorlatinib). https://cima.aemps.es/cima/dochtml/ft/1191355002/FT_1191355002.html.

[29] Informe de Posicionamiento Terapéutico de lorlatinib (Lorviqua) en cáncer de pulmón no microcítico ALK positivo. https://www.aemps.gob.es/medicamentosUsoHumano/informesPublicos/docs/2021/IPT_6-2021-Lorviqua.pdf.

[30] Decisión de ejecución de la Comisión de 6.5.2019. https://ec.europa.eu/health/documents/community-register/2019/20190506144341/dec_144341_es.pdf.

[31] https://www.sanidad.gob.es/profesionales/medicamentos.do?metodo=verDetalle&cn=725704.

[32] Shaw AT, Bauer TM, De Marinis F, Felip E, Goto Y, Liu G (2020). First-line lorlatinib or crizotinib in advanced *ALK*-positive lung cancer. N Engl J Med.

[33] Decisión de ejecución de la Comisión de 27.1.2022. https://ec.europa.eu/health/documents/community-register/2022/20220127154691/dec_154691_es.pdf.

[34] Acuerdos de la reunión de la Comisión Interministerial de precios de los medicamentos. https://www.sanidad.gob.es/profesionales/farmacia/pdf/20221027_ACUERDOS_CIPM_2272.pdf.

[35] Acuerdos de la reunión de la Comisión Interministerial de precios de los medicamentos. https://www.sanidad.gob.es/profesionales/farmacia/pdf/20230202_ACUERDOS_CIPM_230.pdf.

[36] Ficha técnica Vitrakvi (Larotrectinib). https://cima.aemps.es/cima/dochtml/ft/1191385002/FT_1191385002.html.

[37] Tan D, Farago A, Kummar S (2021). MA1109 efficacy and safety of larotrectinib in patients with tropomyosin receptor kinase (TRK) fusion lung cancer. J Thorac Oncol.

[38] Laetsch TW, DuBois SG, Mascarenhas L, Turpin B, Federman N, Albert CM (2018). Larotrectinib for paediatric solid tumours harbouring NTRK gene fusions: phase 1 results from a multicentre, open-label, phase 1/2 study. Lancet Oncol.

[39] Drilon A, Laetsch TW, Kummar S, DuBois SG, Lassen UN, Demetri GD (2018). Efficacy of larotrectinib in TRK fusion-positive cancers in adults and children. N Engl J Med.

[40] Decisión de ejecución de la Comisión de 19.9.2019. https://ec.europa.eu/health/documents/community-register/2019/20190919145810/dec_145810_es.pdf.

[41] https://www.sanidad.gob.es/profesionales/medicamentos.do?metodo=verDetalle&cn=726876.

[42] Acuerdos de la reunión de la Comisión Interministerial de precios de los medicamentos. https://www.sanidad.gob.es/profesionales/farmacia/pdf/ACUERDOS_CIPM_2122_6_MAYO_2021.pdf.

[43] Ficha técnica Rozlytrek (entrectinib). https://cima.aemps.es/cima/dochtml/ft/1201460001/FT_1201460001.html

[44] Informe de Posicionamiento Terapéutico de entrectinib (Rozlytrek) en cáncer de pulmón no microcítico ROS1-positivo. https://www.aemps.gob.es/informa/informes-de-posicionamiento-terapeutico/informe-de-posicionamiento-terapeutico-deentrectinib-rozlytrek-en-cancer-de-pulmon-no-microcitico-ros1-positivo/

[45] Decisión de ejecución de la Comisión de 31.7.2020. https://ec.europa.eu/health/documents/community-register/2020/20200731148534/dec_148534_es.pdf.

[46] https://www.sanidad.gob.es/profesionales/medicamentos.do?metodo=verDetalle&cn=729114.

[47] https://www.sanidad.gob.es/profesionales/farmacia/pdf/ACUERDOS_DE_LA_CIPM_2132_web.pdf.

[48] https://www.aemps.gob.es/medicamentosUsoHumano/informesPublicos/docs/2022/IPT_36-2022-Rozlytrek-CPNM.pdf.

[49] Ficha técnica Retsevmo (selpercatinib). https://cima.aemps.es/cima/dochtml/ft/1201527001/FT_1201527001.html

[50] Drilon A, Oxnard GR, Tan DSW, Loong HHF, Johnson M, Gainor J (2020). Efficacy of selpercatinib in RET Fusion-positive non-small-cell lung cancer. N Engl J Med.

[51] Decisión de ejecución de la Comisión de 11.2.2021. https://ec.europa.eu/health/documents/community-register/2021/20210211150524/dec_150524_es.pdf.

[52] Decisión de ejecución de la Comisión de 21.6.2022. https://ec.europa.eu/health/documents/community-register/2022/20220621156019/dec_156019_es.pdf.

[53] https://www.sanidad.gob.es/profesionales/medicamentos.do?metodo=verDetalle&cn=730406.

[54] Acuerdos de la reunión de la Comisión Interministerial de precios de los medicamentos. https://www.sanidad.gob.es/profesionales/farmacia/pdf/20220707_ACUERDOS_CIPM_225.pdf.

[55] Ficha técnica Gavreto (pralsetinib) https://www.ema.europa.eu/en/documents/product-information/gavreto-epar-product-information_es.pdf.

[56] Gainor JF, Curigliano G, Kim DW, Lee DH, Besse B, Baik CS (2021). Pralsetinib for RET fusion-positive non-small-cell lung cancer (ARROW): a multi-cohort, open-label, phase 1/2 study. Lancet Oncol.

[57] Decisión de ejecución de la Comisión de 18.11.2021. https://ec.europa.eu/health/documents/community-register/2021/20211118153376/dec_153376_es.pdf.

[58] https://www.sanidad.gob.es/profesionales/medicamentos.do?metodo=verDetalle&cn=732331.

[59] Acuerdos de la reunión de la Comisión Interministerial de precios de los medicamentos. https://www.sanidad.gob.es/profesionales/farmacia/pdf/20221027_ACUERDOS_CIPM_227.pdf.

[60] Ficha técnica Rybrevant (amivantamab). https://cima.aemps.es/cima/dochtml/ft/1211594001/FT_1211594001.html.

[61] Park K, Haura EB, Leighl NB, Mitchell P, Shu CA, Girard N (2021). Amivantamab in EGFR exon 20 insertion-mutated non-small-cell lung cancer progressing on platinum chemotherapy: initial results from the CHRYSALIS phase I study. J Clin Oncol.

[62] Decisión de ejecución de la Comisión de 9.12.2021. https://ec.europa.eu/health/documents/community-register/2021/20211209153836/dec_153836_es.pdf.

[63] https://www.sanidad.gob.es/profesionales/medicamentos.do?metodo=verDetalle&cn=732485.

[64] Acuerdos de la reunión de la Comisión Interministerial de precios de los medicamentos. https://www.sanidad.gob.es/profesionales/farmacia/pdf/20221215_ACUERDOS_CIPM_2292.pdf.

[65] Acuerdos de la reunión de la Comisión Interministerial de precios de los medicamentos. https://www.sanidad.gob.es/profesionales/farmacia/pdf/20230223_ACUERDOS_CIPM_231.pdf.

[66] Ficha técnica Tabrecta (capmatinib). https://cima.aemps.es/cima/dochtml/ft/1221650004/FT_1221650004.html.

[67] Wolf J, Seto T, Han J-Y, Reguart N, Garon EB, Groen HJM (2020). Capmatinib in *MET* exon 14-mutated or *MET*-amplified non-small-cell lung cancer. N Engl J Med.

[68] Decisión de ejecución de la Comisión de 20.6.2022. https://ec.europa.eu/health/documents/community-register/2022/20220620155859/dec_155859_es.pdf.

[69] https://www.sanidad.gob.es/profesionales/medicamentos.do?metodo=verDetalle&cn=757148.

[70] Ficha técnica Tepmetko (tepotinib). https://cima.aemps.es/cima/dochtml/ft/1211596001/FT_1211596001.html.

[71] Paik PK, Felip E, Veillon R, Sakai H, Cortot AB, Garassino MC (2020). Tepotinib in non-small-cell lung cancer with *MET* exon 14 skipping mutations. N Engl J Med.

[72] Decisión de ejecución de la Comisión de 16.2.2022. https://ec.europa.eu/health/documents/community-register/2022/20220216154592/dec_154592_es.pdf.

[73] Ficha técnica Lumykras (sotorasib). https://cima.aemps.es/cima/dochtml/ft/1211603001/FT_1211603001.html.

[74] Informe de evaluación SEOM de sotorasib en pacientes con cáncer de pulmón no microcítico (CPNM) avanzado con mutaciones del gen KRAS tipo G12C que han progresado a al menos un tratamiento sistémico previo. https://seom.org/seomcms/images/stories/Informes_SEOM/IEV_SEOM_SOTORASIB.pdf.

[75] Decisión de ejecución de la Comisión de 6.1.2022. https://ec.europa.eu/health/documents/community-register/2022/20220106154095/dec_154095_es.pdf.

[76] https://www.sanidad.gob.es/profesionales/medicamentos.do?metodo=verDetalle&cn=739028.

[77] Planchard D, Besse B, Groen HJM, Souquet PJ, Quoix E, Baik CS (2016). Dabrafenib plus trametinib in patients with previously treated BRAF V600E-mutant metastatic non-small cell lung cancer: an open-label, multicentre phase 2 trial. Lancet Oncol.

[78] Planchard D, Smit EF, Groen HJM, Mazieres J, Besse B, Helland A (2017). Dabrafenib plus trametinib in patients with previously untreated BRAF V600E-mutant metastatic non-small cell lung cancer: an open-label, phase 2 trial. Lancet Oncol.

[79] https://www.ema.europa.eu/en/documents/smop/chmp-post-authorisation-summary-positive-opinion-tafinlar_en.pdf.

[80] https://www.sanidad.gob.es/profesionales/farmacia/pdf/NOTA_INFORMATIVA_DE_LA_CIPM_179_web.pdf.

[81] https://www.sanidad.gob.es/profesionales/medicamentos.do?metodo=verDetalle&cn=699781.

